# A high selective methanol gas sensor based on molecular imprinted Ag-LaFeO_3_ fibers

**DOI:** 10.1038/s41598-017-12337-z

**Published:** 2017-09-21

**Authors:** Qian Rong, Yumin Zhang, Chao Wang, Zhongqi Zhu, Jin Zhang, Qingju Liu

**Affiliations:** School of Materials Science and Engineering, Yunnan Key Laboratory for Micro/nano Materials & Technology, Yunnan University, 650091 Kunming, China

## Abstract

Ag-LaFeO_3_ molecularly imprinted polymers (ALMIPs) were fabricated, which provided special recognition sites to methanol. Then ALMIPs fiber 1, fiber 2 and fiber 3 were prepared using filter paper, silk and carbon fibers template, respectively. Based on the observation of X-ray diffraction (XRD), scanning electron microscope (SEM), transmission electron microscope (TEM), and Nitrogen adsorption surface area analyzer (BET), the structure, morphology and surface area of the fibers were characterized. The ALMIPs fibers (fiber 1, fiber 2 and fiber 3) show excellent selectivity and good response to methanol. The responses to 5 ppm methanol and the optimal operating temperature of ALMIPs fibers are 23.5 and 175 °C (fiber 1), 19.67 and 125 °C (fiber 2), 17.59 and 125 °C (fiber 3), and a lower response (≤10, 3, 2) to other test gases including formaldehyde, acetone, ethanol, ammonia, gasoline and benzene was measured, respectively.

## Introduction

Gas sensor is based on the change of conductivity of gas-sensing materials after adsorbing target gas, so it can measure the concentration of the target gas. The amount of adsorbed oxygen strongly depend on morphology, surface area, structure^1,2^ and grain size^3,4^ of the sensing material. The morphology control is quite beneficial for increasing the amount of adsorbed oxygen and the mobility of oxygen^5^. Methanol is a very hazardous gas and its acute exposure can produce immediate bronchial constriction, narrowing of the airways, and increased pulmonary resistance. Acute exposures to experimental animals have also produced changes in metabolism and irritation to the mucus membranes in eyes. Hence the improvement of a reliable and selective methanol sensor has become essential and urgent. The detection methods include: spectrophotometry^6^, chromatography method^7^, electrochemical method^8^, catalytic luminescence method^9^ and gas sensor^10^. Among them, the advantages of the first four methods are fast detection and high accuracy, but expensive instruments, high cost, large volume and hard to wide application and so on. Gas sensor methods are used to detect toxic gases with a high sensitivity and simple operation. The gas sensors are small devices available at low cost, suitable for real time monitoring and useful for detection of indoor air pollutants. Among many kinds of gas sensors, the oxide semiconductor gas sensors are the mainstream products. Semiconductor gas sensor has been widely favoured for the past twenty years because of its high sensitivity, stable performance, low price, small size, easy to use etc. Ag-LaFeO_3_ (AL) has emerged as one of the most potential materials as gas sensors owing to its large surface area, rich active oxygen lattice, good thermostability, controllable structure and strong reducibility^11^.

Recently, much attention has been paid to the preparation of special morphology complex oxides because of their interesting and distinctive physical and chemical properties which are different from those of conventional bulk materials^12–14^. What’s more, some 3-dimentional porous or hollow structure provides more surface activities, high specific surface area and fast diffusion, which makes gas penetration into sensory layers easy^15,16^. Template method has been proved to be one of the most effective ways to enhance the gas-sensing properties by altering the morphology of gas-sensing materials, such as porous ZnO nanosheets^17^, porous SnO_2_ nanotubes^18^ and poly aniline nanotubes^19^ etc. There are also some other methods being used to obtain special morphologies. Dong *et al*. reported the response of CuO fibers prepared by combustion synthesis to 10 ppm NPA is 29 at operating temperature 200 °C^20^. Han *et al*. reported that the response to 100 ppm methanol of Ce-doped In_2_O_3_ porous nanospheres prepared with hydrothermal method is about 35 at operating temperature 325 °C^21^. Yang *et al*. prepared a-Fe_2_O_3_ hollow spheres based on one-step synthesis and the response to 10 ppm methanol is about 25 at the operating temperature of 280 °C^22^. Tang *et al*. reported SnO_2_-ZnO_2_ nanofiber via step wise-heating electro spinning method present the response to 10 ppm methanol about 7 at operating temperature 250 °C^23^. Vijay *et al*. reported that the response to 10 ppm methanol of mesoporous Ag-doped TiO_2_/SnO_2_ nano-composite prepared with hydrothermal method is about 15 at operating temperature of 275 °C^23^. Liu *et al*. prepared SnO_2_ hollow spheres based on two-step hydrothermal strategy and the response to 10 ppm methanol is about 10 at the operating temperature of 225 °C^24^. In summary, although researchers have gained so many achievements to improve gas sensing performance of materials, but these sensors with special morphologies have the problems of high operating temperature (more than 200 °C) to the low concentration of methanol (10 ppm).

Molecularly imprinted technology (MIT) is to prepare polymers that match the template molecules in space structures and binding sites. In our previous work, formaldehyde was used as a template molecule, acrylamide (AM) was used as a functional monomer and Ag-LaFeO_3_ was used as a cross-linker acquire high response and selective formaldehyde gas sensor^25^. In this study, we use methanol as a template molecule, methacrylate (MAA) as a functional monomer and Ag-LaFeO_3_ was used as a cross-linker to acquire highly selective methanol gas-sensing materials, namely, the Ag-LaFeO_3_ molecularly imprinted polymers (ALMIPs). And then, ALMIPs fibers (ALMFs) are prepared using different template. The methanol sensing properties were studied. Results show that the obtained ALMFs possess good selectivity to methanol at lower operating temperature. Some differences in surface area and structure lead to the diversity in selectivity, response and operating temperature of the ALMFs.

**Figure 1:**
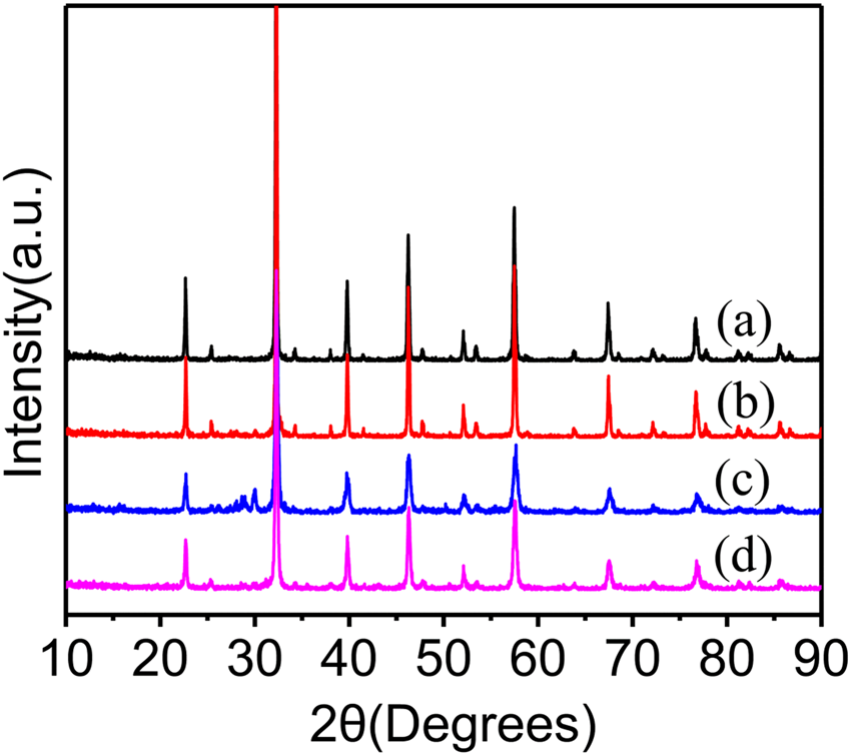
XRD patterns of (**a**) ALMIPs powder, (**b**) ALMF-1, (**c**) ALMF-2 and (**d**) ALMF-3.

X-ray powder diffraction patterns of the prepared ALMFs are shown in Fig. 1. The patterns in Fig. 1 indicate that the structure of ALMFs is orthogonal perovskite, which includes only one phase of LaFeO_3_. No impure peaks were observed in the XRD patterns, indicating high purity of the samples. In the XRD patterns of ALMF-2, the diffraction peaks located at 2θ = 27 and 29° are observed, this is because the purchased silk contains a small amount of impurities, no completely removed after sintering. The filter paper, carbon fiber, functional monomer and initiator are removed after sintering; only some functional group is left on the cross-linker^26^, which cannot be detected.

**Figure 2:**
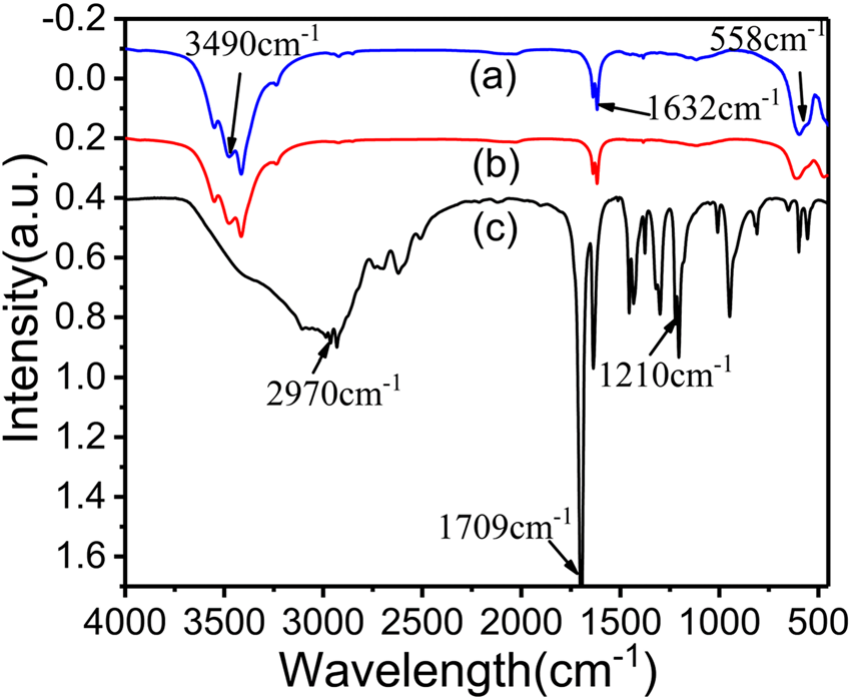
Infrared spectra of (**a**) Ag-LaFeO_3_ cross-linker, (**b**) ALMF-1 and (**c**) MAA.

Figure 2 shows the FT-IR spectra of cross-linker (Ag-LaFeO_3_), ALMF-1 and the functional monomer Methacrylate (MAA) in the range of 450–4000 cm^−1^. In the curve of (a), the peaks around 558 cm^−1^, and 3490 cm^−1^ indicate Fe-O vibrations, and the stretching vibration of O-H^27^ of H_2_O in air respectively, and the peaks around 1632 cm^−1^ are attributed to the La-O vibrations^28^. Comparing the curves of (b) and (c), the disappearance of the relatively strong peak of the C = O stretching vibration (1709 cm^−1^) and C-O the stretching vibration (1210 cm^−1^)^29^ in amidogen of ALMF-1 suggest the successful interaction between Ag-LaFeO_3_ and MAA, and the interaction should be ascribed to the coordination between amidogen groups in MAA and La in Ag-LaFeO_3_^30^.

**Figure 3:**
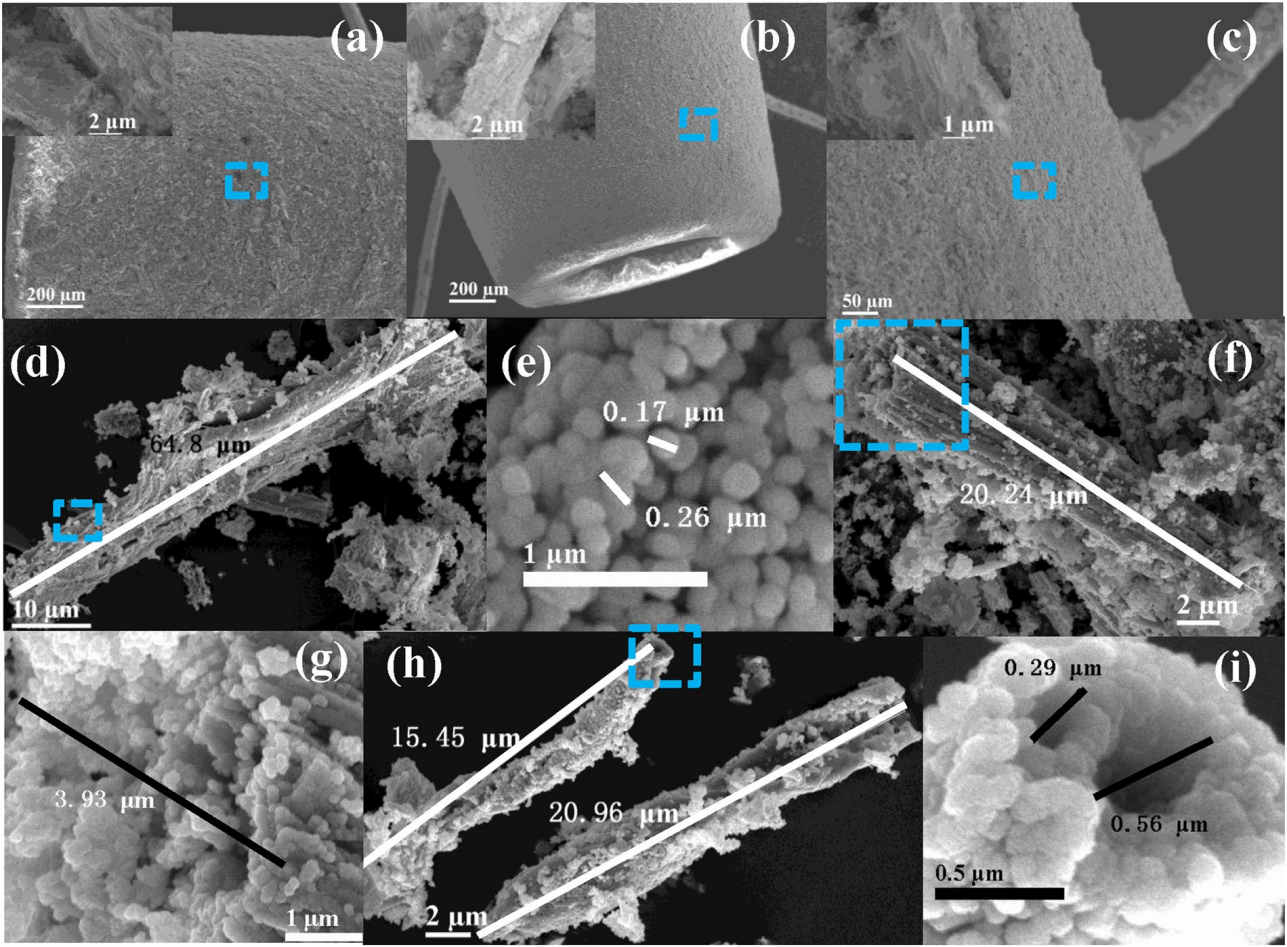
SEM images of the ALMFs, (**a**), (**b**) and (**c**) SEM image of ceramic tube of ALMF-1, ALMF-2 and ALMF-3; (**d**), (**f**) and (**h**) ALMF-1, ALMF-2 and ALMF-3; (**e**), (**g**) and (**i**) high resolution morphology image of selected areas of ALMF-1, ALMF-2 and ALMF-3.

Figure 3 shows the SEM images of the ALMFs samples. Figure 3a,b and c are the low magnification SEM of the ALMFs on the surface of Al_2_O_3_ ceramic tube, it can be seen that a large number of ALMFs are adhered to the surface of Al_2_O_3_ ceramic tube and form a thick film in the inset in Fig. 3a,b and c. Figure 3d reveals that the cellulosic structure of ALMF-1 was at length of 64.8 μm. ALMF-1 comprises of numerous micron-particles with the diameter about 0.21 μm which shows in Fig. 3e. Figure 3f shows the SEM image of the ALMF-2 samples, it shows that ALMF-2 is a fiber structure and with the length of 20.24 μm. Figure 3g shows the high resolution morphology image of the selected area in Fig. 3f of the ALMF-2 samples, it can be seen that ALMF-3 is a solid fiber structure and its section diameter is 3.93 μm. Figure 3h reveals that the cellulosic structure of ALMF-3 with the length of about 18.2 μm. The ALMF-3 is hollow cellulosic structure, the diameter of the voids is about 0.42 μm as show in Fig. 3i which could be a very efficient gas passage. All of above indicate that the ALMF-1 has relatively larger surface area due to its long length and a large number of particles than ALMF-2 and ALMF-3.

**Figure 4:**
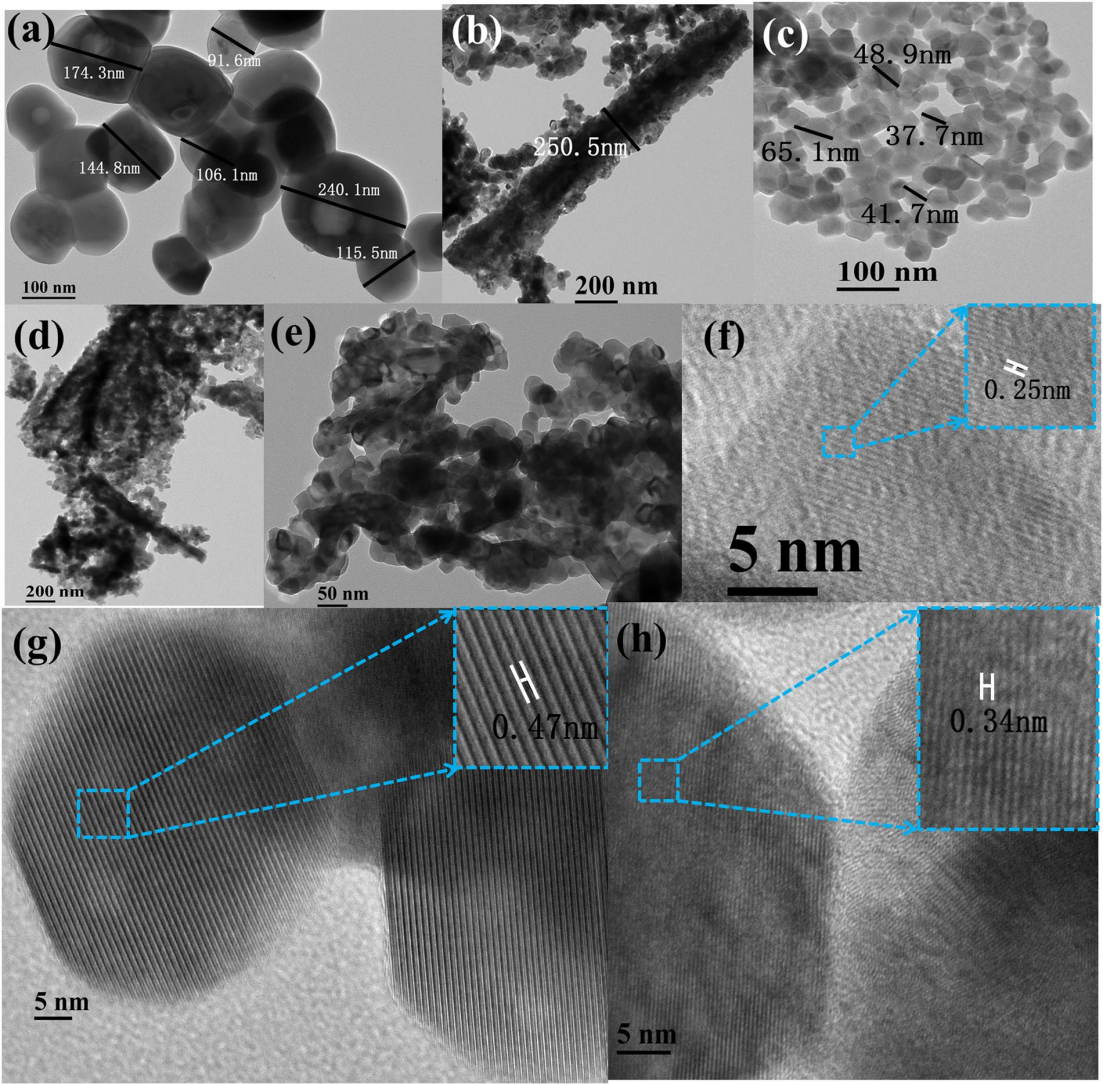
TEM image of as-prepared ALMFs, (**a**) ALMF-1, (**b**) and (**c**) ALMF-2, (**d**) and (**e**) ALMF-3, (**f**), (**g**) and (**h**) high resolution crystal lattice of ALMF-1, ALMF-2 and ALMF-3.

TEM observations were also conducted to provide insight into more detailed structural features of the ALMF-1, ALMF-2 and ALMF-3. Figure 4 shows typical TEM images of ALMFs. Figure 4a displays a low magnification TEM image of the as-prepared ALMF-1, which illustrated that the sample was made up of micro-particles with sizes of 88–245 nm. The cellulosic feature of ALMF-1 can also be clearly identified. High-resolution transmission electron microscopy (HRTEM) measurement was applied to provide the internal microstructure information on the as-prepared ALMF-1. The lattice fringes can be apparently observed from Fig. 4f, and value of the inter-planar spacing between the adjacent lattices is 0.25 nm. Figure 4b and c show a structure of the ALMF-2 in low-magnification TEM image which illustrates that the ALMF-2 are composed of irregularly shaped particles with the sizes ranging from 30 to 65.1 nm and well dispersed. The adjacent lattice of ALMF-2 is 0.47 nm as showed in Fig. 4g. The crystal lattice fringes are obvious, which means the crystallinity of this sample is good. TEM image of the ALMF-3 in Fig. 4d has also confirmed their cellulosic feature. Figure 4e shows that ALMF-3 is assembled from a large amount of interconnected nanoparticles, particle dispersion is not good. The lattice image of a nanoparticle of the ALMF-3 shown in Fig. 4h, observed lattice perpendicular is about 0.34 nm. In summary, ALMFs all compose with small particles and those particles are different in size, shape, dispersion and crystallinity, which may lead different surface area and response, selectivity and operating temperature. Different templates allow the fibers to have different exposed facets^20,23^, different adjacent lattice results in different properties. The crystal lattice fringes of ALMFs are obvious, but it can be seen that the crystallinity of ALMF-2 is better than that of ALMF-1 and ALMF-3.

**Figure 5:**
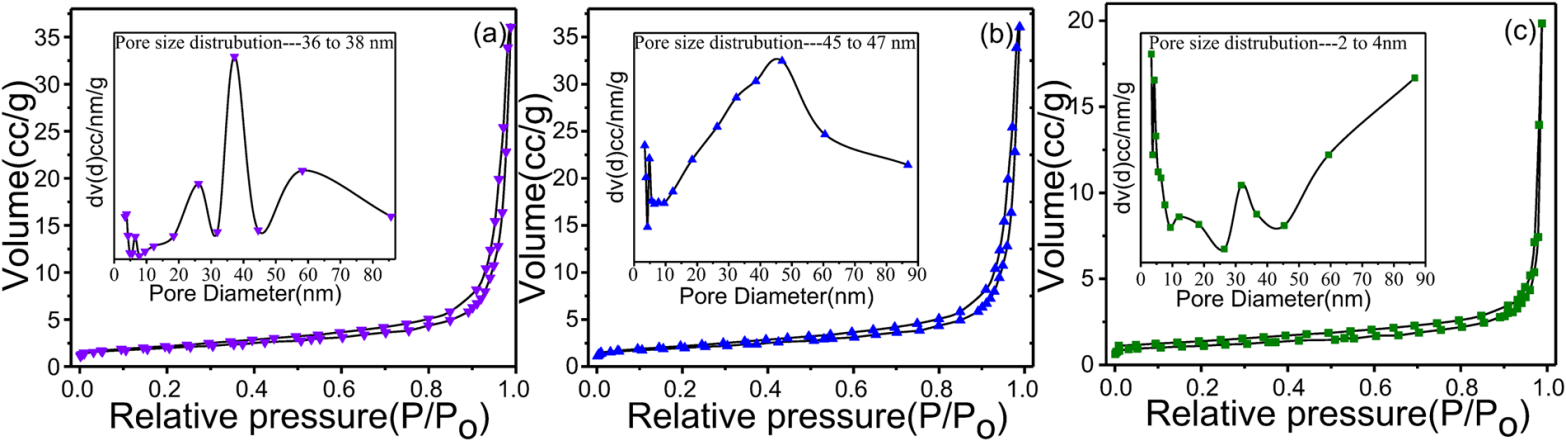
The corresponding pore size distribution curve (inset) of (**a**) ALMF-1, (**b**) ALMF-2 and (**c**) ALMF-3.

The structure of the cellulose and the specific surface area of the samples are examined by the nitrogen adsorption-desorption isotherms and the corresponding BJH pore size distributions curves. The results are shown in Fig. 5 that all three samples show obvious hysteresis loop because of the existence of pores. Meanwhile, the pore-size distributions are calculated by the BJH method, as observed in inset of Fig. 5 ^31^. The ALMF-1 possesses higher surface area is 11.4 m^2^g^−1^ and exhibit hysteresis loops at the P/P_0_ ranges between 0.003 and 0.99 with a pore size distribution of 36–38 nm. Surface area of the ALMF-2 is 6.9 m^2^g^−1^ and the P/P_0_ range 0.003–0.99 with a pore size distribution of 45–47 nm. Surface area of the ALMF-3 is 3.92 m^2^g^−1^ and exhibit hysteresis in the P/P_0_ ranges 0.005–0.99 with a pore size distribution of 2–4 nm. The surface areas of the different ALMFs prepared by the sol-gel served with template method in the present work are higher than the previously results of hydrothermal method of 2.5 m^2^g^−1^ ^32^. The ALMF-1 has larger surface area than that of the other ALMFs, which is good accordance with the results of SEM and TEM. That suggests the as-prepared ALMFs possess high response and selectivity because of the larger surface area.

**Figure 6:**
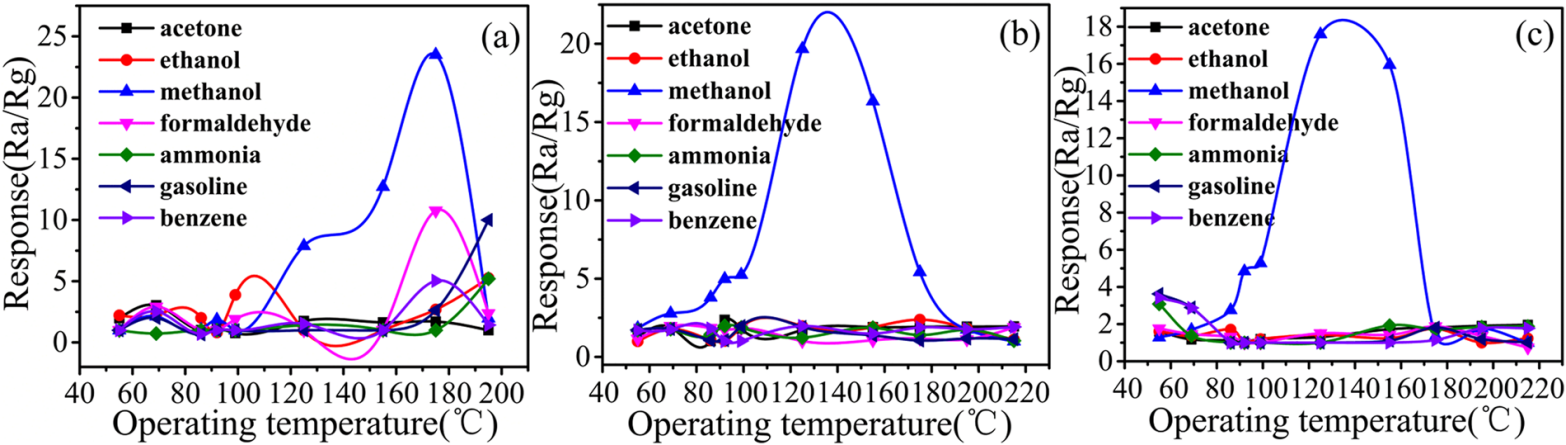
Response curves of ALMFs sensors, (**a**) ALMF-1, (**b**) ALMF-2 and (**c**) ALMF-3 towards different tested gases with 5 ppm concentration.

The gas response S was defined as the ratio of the electrical resistance in gas (Rg) to that in air (Ra). To investigate the gas-sensing properties of ALMF-1, ALMF-2 and ALMF-3, the response of 5 ppm toluene, ammonia, acetone, gasoline and methanol were tested under different operating temperatures (Fig. 6a–c). Figure 6a shows the gas sensing properties of ALMF-1 for different test gases. It can be seen that this sensor exhibits a response of 23.5 to methanol at the operating temperature of 175 °C while shows obvious lower response (≤10) to the other test gases. In Fig. 6b, the best response to 5 ppm methanol based on ALMF-2 is 19.67 at 125 °C but for the other test gases, the highest response is all lower than 3.0. Figure 6c shows the gas sensing properties of ALMF-3, it can be seen that the best response to methanol is 17.59 at 125 °C, while shows a distinct lower response (<2) to the other test gases. For the three samples, ALMF-1 exhibits the highest response (23.5) than ALMF-2 and ALMF-3 on account of its largest surface area. Compared with other semiconducting metal oxides reported in literatures^12–14^, ALMFs shows lower operating temperature. The mechanism of above phenomenon can be discussed as follow. The BET test revealed that the specific surface area of the ALMFs is relatively larger, which is benefit for absorbing the methanol molecules and proceeding gas-sensing reaction. Compared with methanol and toluene, gasoline and formaldehyde are much reductive. And ALMF-1 possesses the largest specific surface area among the 3 ALMF samples and thus has the most absorbed oxygen. When gasoline and formaldehyde react with ALMF-1, resistance change is the most obvious. So, ALMF-1 exhibit higher response to gasoline and formaldehyde and hence the selectivity of ALMF-1 is not as good as ALMF-2 and ALMF-3. And the cellulose structure and small lattice spacing of material could easily transport the electrons formed from the gas-sensing reaction, thus enhanced the carrier mobility of the sensor, finally result in lower operating temperature^33^. In the prepared Ag-LaFeO_3_ sample, some Ag in the form of single matter as the catalyzer mixes in the matrix. Some of them are filled between the grains of the matrix to decrease the contact potential barrier and enhance the interfacial effect, which leads to lower resistance and finally results in lower operating temperature. So ALMFs has a lower operating temperature compared with LaFeO_3_. The responses of ALMFs to methanol are much higher than the responses reported in literature, as shown in Table 1 ^34–48^. Operating temperature is generally high reported in literatures, but operating temperature of the sensor based on ALMFs respective is 175 °C, 125 °C and 125 °C in this work.

**Table 1:** Comparison of the sensing performances of multiple materials based gas sensors toward methanol.

Materials	Response	Optimal temperature (°C)	References
Porous In_2_O_3_ nanobelts	9.5 (20 ppm)	370	34
SnO_2_-ZnO nanofibers	8.5 (10 ppm)	350	35
Ce-In_2_O_3_ nanospheres	35.2 (100 ppm)	320	36
Cu fibers	35 (100 ppm)	325	21
α-Fe_2_O_3_ polyhedral crystals	2.5 (50 ppm)	340	37
ZnO/SnO_2_ nanostructures	9.6 (100 ppm)	300	38
α-Fe_2_O_3_	25 (10 ppm)	280	22
Au-decorated ZnO	7 (50 ppm)	300	39
SnO_2_-ZnO nanofiber	7 (10 ppm)	250	54
Pd_0.5_Pd_3_O_4_-ZnO	10.5 (50 ppm)	260	40
CdS-SnO_2_	70 (5000 ppm)	200	41
Zn-SnO_2_ nanorods clusters	59 (50 ppm)	270	42
Ag-TiO_2_/SnO_2_	15 (10 ppm)	275	23
α-Fe_2_O_3_ discoid crystals	6.4 (100 ppm)	250	43
Al-ZnO thin films	1.79 (500 ppm)	275	44
WO_3_	24 (100 ppm)	260	45
La_0.8_Pd_0.2_FeO_3_	146.6 (400 ppm)	230	46
SnO_2_ hollow spheres	10 (10 ppm)	225	24
Macroporous LaFeO_3_	96 (100 ppm)	190	47
CuO thin film	0.12 (500 ppm)	350	48
ALMF-1	23.5 (5 ppm)	175	This work
ALMF-2	19.67 (5 ppm)	125	
ALMF-3	17.59 (5 ppm)	125	

**Figure 7:**
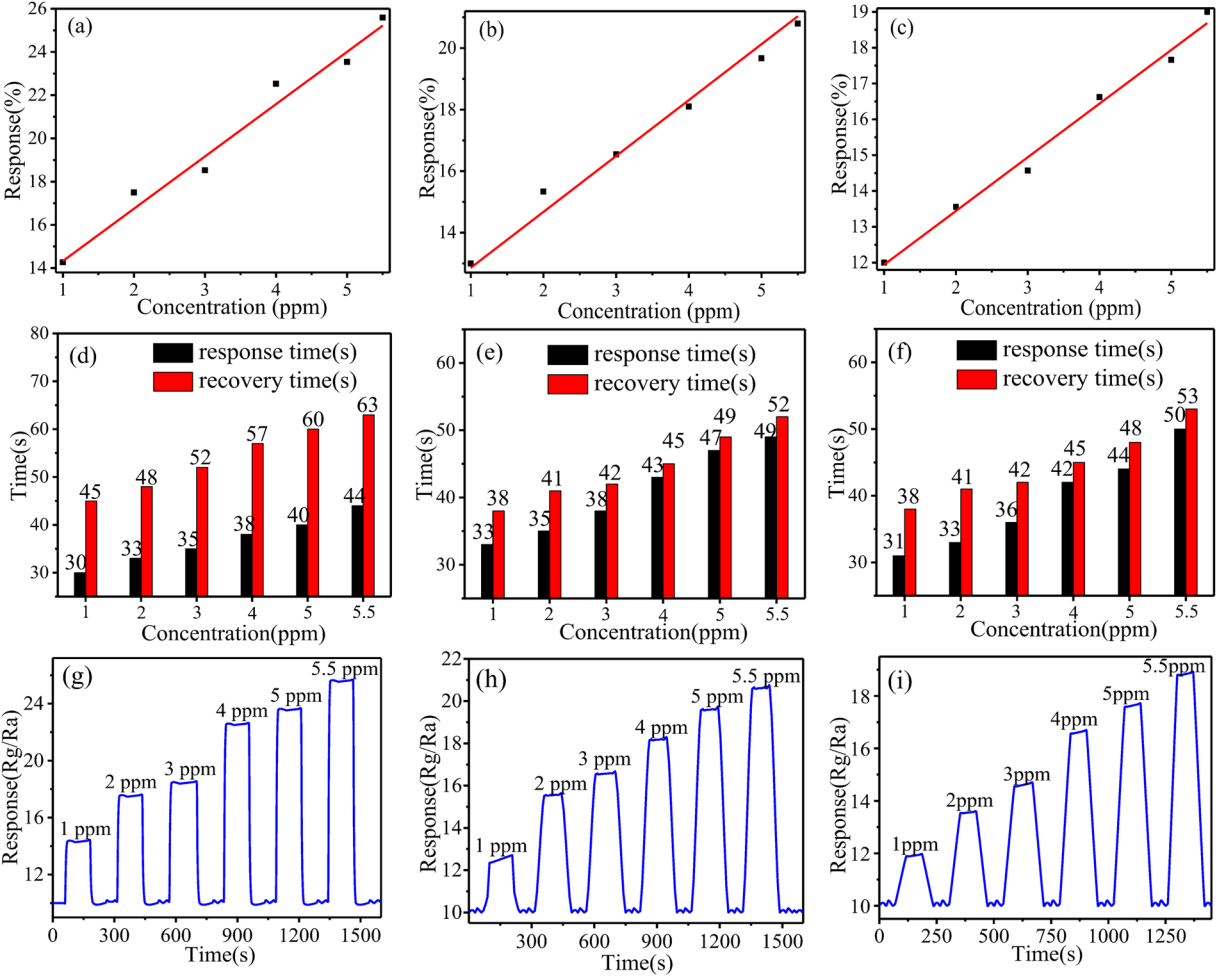
The relationship of response to different concentrations methanol gas based on (**a**) ALMF-1; 175 °C, (**b**) ALMF-2; 125 °C and (**c**) ALMF-3; 125 °C; response-recovery times to different concentrations of methanol gas based on (**d**) ALMF-1; 175 °C, (**e**) ALMF-2; 125 °C and (**f**) ALMF-3; 125 °C; dynamic response of the ALMFs sensor to methanol with increasing concentrations based on (**g**) ALMF-1; 175 °C, (**h**) ALMF-2; 125 °C and (**i**) ALMF-3; 125 °C.

The relationship between response and methanol concentration, as well as the response-recovery time, were investigated with the sensor based on ALMF-1, ALMF-2 and ALMF-3 at the operating temperature of 175 °C, 125 °C and 125 °C, respectively was illustrated in Fig. 7. It can be seen that the response increasing for ALMF-1, ALMF-2 and ALMF-3 sample are near linear with the concentration of the methanol at the operating temperature of 175 °C, 125 °C and 125 °C, respectively as showed in Fig. 7a–c, indicating that the sensor can be used as a continuous real-time monitoring at lower concentration of methanol at the optimum operating temperature. The response and recovery times are defined as the time taken by the sensor to achieve 90% of the initial equilibrium resistance change in the adsorption and desorption processes, respectively. The value of the response and recovery time to 1 ppm methanol based on ALMF-1, ALMF-2 and ALMF-3 are 30 s and 45 s, 33 s and 38 s, 31 s and 38 s, respectively. The response and recovery time to methanol with other concentration see Fig. 7d–f. Figure 7g–i show the dynamic response and recovery characteristic of the ALMFs sensor to different concentration of methanol at 175 °C (ALMF-1), 125 °C (ALMF-2) and 125 °C (ALMF-3), respectively. It is found that the response of the sensor is enhanced with the increase of methanol concentration.

**Figure 8:**
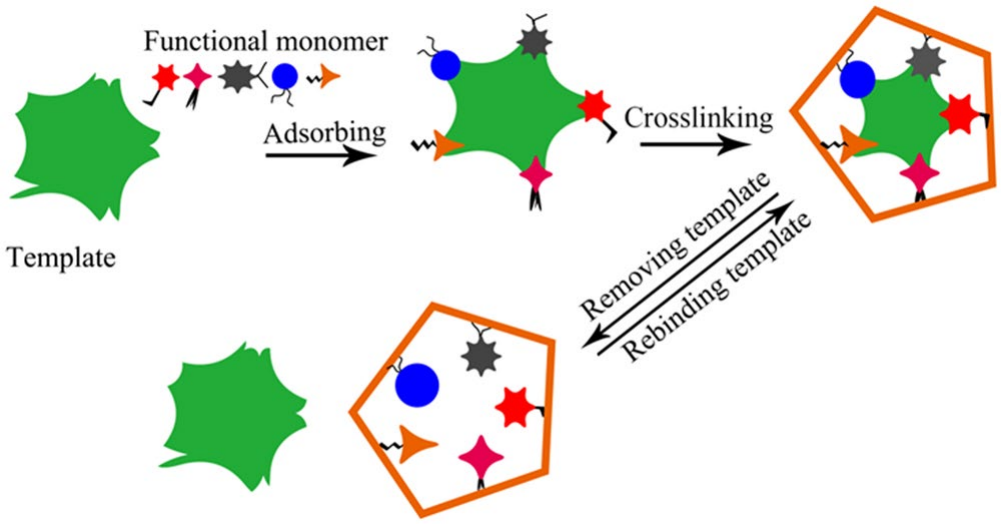
Schematic diagram of gas-sensing mechanism of the MINs.

MAA has been used as a “universal” functional monomer due to its hydrogen bond donor and acceptor characteristics^49^. Thus, when the template molecule (methanol) mixed with functional monomers (MAA), methanol is allowed to interact with MAA via hydrogen-bond, and then a methanol-MAA complex is formed^50^, as shown in Fig. 8. A cross-linker is used to fix functional monomers around template molecules, there by forming a highly cross-linked rigid polymer even after the removal of templates, a mass of the imprinted caves capable of recognizing and re-binding the methanol is left^51^. The caves are complementary to the methanol in size, shape, and position of the functional groups^51^, so the sensors show good selectivity to methanol. This also indicates that molecularly imprinted technique is a practical method for improving the selectivity of the gas sensor.

After molecular imprinted polymerization with MAA, the gas sensing mechanism of ALMFs is similar to that of LaFeO_3_, only with markedly increase of selectivity and response to methanol. LaFeO_3_ is a typical P-type semiconductor, and the gas sensing mechanism is based on the changes of the resistance before and after being exposed to the test gas^51^. In the process of calcining at high temperature they lost the lanthanum atoms at the crank points of cells and left lanthanum vacancies, the conductivities of LaFeO_3_ is due to the ionizing of lanthanum vacancies^40,52^. When sensors exposed to the air, O_2_ was adsorbed on LaFeO_3_ surface and ionized to and by capturing free electrons of the particles, which would trap electrons from the body of LaFeO_3_ due to the strong electronegativity of the oxygen atom and produced chemisorbed oxygen. So the concentration of holes in valence band increased and the resistance of materials decreased due to the increasing concentration of available carrier^53^. On the other hand, when CH_3_OH is allowed to enter the sensor, it reacts with the adsorbed oxygen to form CO_2_ and H_2_O and releasing the electrons. This process results in the thickening of the space-charge layer, thus increasing the potential barrier and decreasing the current, and also the resistance of the sensor is increased. This mechanism can be expressed as following:

1 $${{\rm{V}}}_{\mathrm{La}}^{{\rm{X}}}\to {{\rm{V}}}_{\mathrm{La}}^{{\prime\prime\prime}}+3{{\rm{h}}}^{\cdot }$$

2 $${{\rm{O}}}_{2({\rm{ads}})}+{{\rm{e}}}^{-}\leftrightarrow {{\rm{O}}}_{2({\rm{ads}})\,}^{-}$$

3 $${{\rm{O}}}_{2({\rm{ads}})}^{-}+{{\rm{e}}}^{-}\leftrightarrow 2{{\rm{O}}}_{({\rm{ads}})}^{-}$$

4 $${{\rm{O}}}_{({\rm{ads}})}^{-}+{{\rm{e}}}^{-}\leftrightarrow {{\rm{O}}}_{({\rm{ads}})}^{2-}$$

5 $${{\rm{CH}}}_{3}{{\rm{OH}}}_{({\rm{ads}})}+2{{\rm{O}}}_{({\rm{ads}})}^{2-}\leftrightarrow {{\rm{CO}}}_{2}+{{\rm{H}}}_{2}{\rm{O}}+4{{\rm{e}}}^{-}$$

In summary, ALMFs were successfully fabricated by combining sol-gel method, molecular imprinting technique and template method. It is found that the as-prepared fibers are orthogonal perovskite with high crystallinity and purity. The ALMFs exhibit cellulosic structure with a hollow or solid rod consisting of single fiber. The specific surface areas of the ALMFs are 11.4 m^2^g^−1^, 6.9 m^2^g^−1^ and 3.92 m^2^g^−1^ respectively. The responses of the ALMFs gas sensor for 5 ppm methanol were higher than the other test gases. The response and operating temperature of ALMF-1, ALMF-2 and ALMF-3 are respectively 23.5 and 175 °C, 19.67 and 125 °C, 17.59 and 125 °C, and the response time and recovery time are 40 s and 60 s, 47 s and 49 s, 44 s and 48 s, respectively. To sum up, the results show that the gas sensor exhibits excellent sensing performances towards methanol, and truly realized low limit, high response and high selectivity of detection.

## Methods

### Preparation of ALMIPs

All the chemical reagents used in the experiments were obtained from commercial sources as guaranteed grade reagents and used without further purification. Silver nitrate (AgNO_3_), lanthanum nitrate (La(NO_3_) • 6H_2_O), ferric nitrate (Fe(NO_3_)_3_ • 9H_2_O), citric acid (C_6_H_8_O_7_ • H_2_O) and polyethylene glycol were of analytical grade. The Ag-LaFeO_3_ perovskite precursors were prepared based sol-gel method. In a typical procedure, 20 mmol of citric acid, 9.9 mmol of La(NO_3_)_3_ • 6H_2_O and 10 mmol of Fe(NO_3_)_3_ • 9H_2_O were first dissolved in 90 mL of deionized water as solution A. 0.1 mmol silver nitrate were dissolved in 10 mL distilled water and added to solution A drop by drop (12 drop per min), and then polyethylene glycol (PEG) was added. The final mixed solution was kept under stirring at 80 °C for 8 h, the mixture turned into a transparent and homogeneous yellow sol. And then was put in a microwave chemical device (CEM, USA) at 75 °C for 2 h, and the sol of Ag-LaFeO_3_ was formed, the sol then used as cross-linker in the molecular imprinting process. The final sol was called solution B in the following text. And then methanol was used as template, methacrylate (MAA) was used as functional monomer, azodiisobutyronitrile (AIBN) was used as initiator. 1.0 mmol methanol mixed with 4 mmol methacrylate, the mixed solution was treated by ultrasonic concussion for 30 min, and allowed to stand for 8 h, named solution C. Then, 1.0 mmol AIBN was dissolved in 20 mL methanol and mixed with solution C and solution B. The final mixture was treated by ultrasonic concussion for 30 min. Finally, stirred at 50 °C for 12 h with the protection of nitrogen and circulating water, the gel of ALMIPs were prepared.

Preparation of ALMFs:

1. Preparation of ALMIPs fiber 1 (ALMF-1): filter paper template was immersed into the above ALMIPs gel and kept overnight, followed by drying at room temperature. The template was burned out by calcination in air at 800 °C for 2 h to obtain ALMF-1.
2. Preparation of ALMIPs fiber 2 (ALMF-2): silk template was immersed into the above ALMIPs gel and kept overnight, and then dried at room temperature. The template was burned out by calcination in air at 800 °C for 2 h to obtain ALMF-2.
3. Preparation of ALMIPs fiber 3 (ALMF-3): the cotton was burned in a furnace at 1000 °C for 30 min, then obtained carbon fiber after cooling to room temperature. Carbon fiber template was immersed into the above ALMIPs gel and kept overnight, then dried at room temperature. The template was burned out by calcination in air at 800 °C for 2 h to obtain ALMF-3.

### Fabrication of sensors

The prepared ALMFs were mixed with distilled water and ground to form a paste, which was subsequently printed onto an alumina tube. There are two Au electrodes placed at both sides of the tube. The length of the alumina tube is 4 mm and the diameter is 1.2 mm. In order to improve their stability and repeatability, the gas sensors were aged at 150 °C for 170 h in air. The gas response was defined as the ratio of the electrical resistance in gas (Rg) to that in air (Ra). The gas-sensing properties were tested using a WS-30A gas senor tester.

### Characterization

The X-Ray Diffraction (XRD) patterns were obtained for the phase identification with a D/max23 diffractometer using Cu Kα radiation (λ = 1.540 Å). The accelerating voltage was 35 kV and the applied current was 25 mA, and the sample was scanned from 10° to 90° (2θ) in steps of 0.02°. The functional group was identified using a Fourier transform infrared spectrometer (FTIR, FTS-40) and the sample was scanned from 450 cm^−1^ to 4000 cm^−1^ by a KBr pellet method. The particle morphology and internal microstructure information of the sample were tested using transmission electron microscope TEM (S-3400N) and scanning electron microscope (SEM). The specific surface area of the sample is tested by the Quadrasorb-evo instrument of the Quantachrome company^55^.
